# Foundation-Model-Driven Skin Lesion Segmentation and Classification Using SAM-Adapters and Vision Transformers

**DOI:** 10.3390/diagnostics16030468

**Published:** 2026-02-03

**Authors:** Faisal Binzagr, Majed Hariri

**Affiliations:** 1 Department of Computer Science, Faculty of Computing and Information Technology-Rabigh, King Abdulaziz University Rabigh, Jeddah 21589, Saudi Arabia; 2 Faculty of Computer and Information System, Islamic University of Madinah, Madinah 42351, Saudi Arabia; majedoh@iu.edu.sa

**Keywords:** foundation models, segment anything model, vision transformer, skin lesion segmentation, skin cancer classification, dermoscopic image analysis, melanoma detection

## Abstract

**Background:** The precise segmentation and classification of dermoscopic images remain prominent obstacles in automated skin cancer evaluation due, in part, to variability in lesions, low-contrast borders, and additional artifacts in the background. There have been recent developments in foundation models, with a particular emphasis on the Segment Anything Model (SAM)—these models exhibit strong generalization potential but require domain-specific adaptation to function effectively in medical imaging. The advent of new architectures, particularly Vision Transformers (ViTs), expands the means of implementing robust lesion identification; however, their strengths are limited without spatial priors. **Methods:** The proposed study lays out an integrated foundation-model-based framework that utilizes SAM-Adapter-fine-tuning for lesion segmentation and a ViT-based classifier that incorporates lesion-specific cropping derived from segmentation and cross-attention fusion. The SAM encoder is kept frozen while lightweight adapters are fine-tuned only, to introduce skin surface-specific capacity. Segmentation priors are incorporated during the classification stage through fusion with patch-embeddings from the images, creating lesion-centric reasoning. The entire pipeline is trained using a joint multi-task approach using data from the ISIC 2018, HAM10000, and PH2 datasets. **Results:** From extensive experimentation, the proposed method outperforms the state-of-the-art segmentation and classification across the dataset. On the ISIC 2018 dataset, it achieves a Dice score of 94.27% for segmentation and an accuracy of 95.88% for classification performance. On PH2, a Dice score of 95.62% is achieved, and for HAM10000, an accuracy of 96.37% is achieved. Several ablation analyses confirm that both the SAM-Adapters and lesion-specific cropping and cross-attention fusion contribute substantially to performance. Paired *t*-tests are used to confirm statistical significance for all the previously stated measures where improvements over strong baselines indicate a p<0.01 for most comparisons and with large effect sizes. **Conclusions:** The results indicate that the combination of prior segmentation from foundation models, plus transformer-based classification, consistently and reliably improves the quality of lesion boundaries and diagnosis accuracy. Thus, the proposed SAM-ViT framework demonstrates a robust, generalizable, and lesion-centric automated dermoscopic analysis, and represents a promising initial step towards clinically deployable skin cancer decision-support system. Next steps will include model compression, improved pseudo-mask refinement and evaluation on real-world multi-center clinical cohorts.

## 1. Introduction

Skin cancer is among the most common and fastest growing types of cancer in the world, with melanoma accounting for most of the skin cancer-related deaths due to its aggressive nature and strong metastatic capacity [1,2]. Thus, early detection and accurate diagnosis are critical to improving the patient outcome, as there is a significant difference in survival curbing melanoma when identified at localized stages [3]. Dermoscopy is a newly developed non-invasive imaging method to visualize subsurface skin structures that would otherwise remain undetected with normal lighting. The implementation of dermoscopy allows an enhanced visualization to identify patterns such as pigment networks, globules, and streaks that would not be seen without dermoscopy [4,5]. A drawback of dermoscopy is that manual evaluations remain purely subjective and rely on the clinician’s skill, interobserver variance and complexity of the lesion being assessed; therefore, computer-aided diagnostic systems have been developed [6,7].

The use of deep learning has recently advanced automated segmentation and classification models for skin lesions. Convolutional neural networks (CNNs) have shown a strong capability for the detection of edges, local texture patterns, and structural irregularities associated with malignancies [8,9,10]. Vision Transformers (ViTs) and hybrid CNN–Transformer architectures have contributed to the feature representation across long-range dependencies and global contextual information [11,12,13]. These models achieve state-of-the-art performance on public dermoscopy datasets such as ISIC 2018, HAM10000, and PH2 [14,15,16]. However, a number of challenges remain.

To begin, the precise segmentation of lesions is still challenging because of low contrast, unclear edges, hair artifacts, lighting changes, and variability in lesion shape [17,18]. Even though segmenters using CNNs and transformers have shown improved segmentation, behavior is generally degraded when applying to external datasets as a consequence of domain shift due to acquisition device, patient selection, and imaging protocols [19,20]. Second, classification models often operate on full-frame dermoscopy images which do not use lesion boundaries, and thus exploit features that contain contamination from the information in irrelevant background regions, such as skin texture, vignetting, rulers, gel bubbles, and color calibration artifacts [21,22]. Third, segmentation and classification typically are approached as distinct tasks, which limits optimal feature exploitation, and overall robustness of the diagnostic [23,24]. Recent advances in medical imaging have explored hyperspectral representations for skin cancer analysis, such as the work of Lin et al. [25], which demonstrated enhanced classification performance using machine learning on hyperspectral data, and Huang et al. [26], who proposed a snapshot-based hyperspectral conversion framework combined with YOLO models for lesion detection.

The emergence of foundation models, particularly the Segment Anything Model (SAM), introduces new opportunities for dermoscopic analysis. SAM provides powerful general-purpose segmentation capabilities learned from billions of masks across diverse visual domains [27]. However, its adaptation to medical imaging requires task-specific fine-tuning and domain-aware integration strategies [28,29]. Furthermore, ViTs have demonstrated strong potential for medical image classification due to their ability to encode global dependencies, but few studies have explored their synergy with foundation-model-derived spatial priors [30,31]. Integrating SAM-based lesion delineation with ViT-based classification therefore represents a promising direction for advancing automated skin cancer diagnosis.

Although significant advances have been made in the development and application of dermoscopic images, the current methods remain inadequate for analyzing dermoscopic images in non-standardized environments or heterogeneous datasets due to major limitations. For example, there are no effective cross-domain generalization capabilities for dermoscopic segmentation models and classification workflows tend to neglect the use of lesion boundary features resulting in the creation of artefacts from the background. Segmentation and classification are treated mostly as individual tasks and do not benefit from mutual reinforcement of spatial/semantic information. Thus, it is critical to develop a framework for dermoscopic image analysis which combines the spatial priors of the lesion with the diagnostic reasoning of transformers, creating a single, model-oriented foundation for dermoscopic image analysis.

The main contributions of this study can be summarized as follows:

- We propose a foundation-model-driven dermoscopic analysis framework based on SAM that utilizes the use of lightweight, parameter efficient adapters to produce robust segmentation results of malignant lesions.
- We introduce a novel segmentation based image classification strategy through use of the Vision Transformer, integrating lesion priors via image cropping to the lesion region(s) and via cross-attention fusion.
- We develop a unified joint optimization strategy allowing for the simultaneous optimization of segmentation and classification tasks; we provide empirical evidence that this strategic approach results in consistent performance improvements across ISIC 2018, PH2 and HAM10000 datasets.

The remainder of this paper is organized as follows. Section 2 reviews recent developments in dermoscopic image segmentation and classification, including the gaps and limitations of the current state of the art. Section 3 elaborates on the proposed framework, which uses foundation models, to perform segmentation and classification. Section 4 provides experimental results, including quantitative comparisons and ablation studies. Finally, Section 5 discusses conclusions and opportunities for future research.

## 2. Related Work

Deep learning has changed the landscape of automated dermoscopic image analysis by facilitating more efficient feature extraction, boundary delineation, and lesion classification across large-scale dermoscopy datasets. The dermatology community has witnessed the evolution of research from standard convolutional neural networks, utilizing datasets such as ISIC 2018, PH2, and HAM10000, to use hybrid pipelines incorporating transformers, multiscale feature extractors, adversarial optimization, and semi-supervised learning. Collectively, these efforts have demonstrated an evolution in developing architectures that take into account both local texture patterns as well as global contextual structures, both of which are fundamental to the reliability of automated melanoma screening systems.

In the area of skin lesion segmentation with the ISIC 2018 dataset, several contemporary models have indicated high performance simply from the innovation of the architecture. FAT-Net was introduced as a feature-adaptive hybrid that had a CNN encoder capturing local edge information with a transformer branch modeling long-range dependencies. Failure of multiscale fusion and attention modulation allowed FAT-Net to achieve strong adaptation to lesion boundaries, fundamentally outperforming other models by obtaining a reported Dice coefficient of 91 percent and Jaccard index 82 percent [32]. Adversarial methods have also been successful; the Efficient-GAN (EGAN) model introduced squeeze–excitation blocks in the generator and a shape-aware discriminator that encourages anatomical consistency in each mask predicted by the model, obtaining a Dice score of 90.1% and IoU accuracy of 83.6% [33]. Semi-supervised learning further improved results on ISIC 2018 through teacher–student training as demonstrated by a DeepLabV3-based self-training framework that leverages unlabeled dermoscopy images from additional ISIC cohorts, reporting a mean IoU of 88% [34]. More advanced CNN–Transformer hybrids, such as MRP-UNet, integrate multiscale input fusion, Res2-SE blocks, and pyramid dilated convolutions to address scale variability and texture heterogeneity in lesions. MRP-UNet achieves a Dice of 92.36% and Jaccard of 91.28% [35]. Texture-aware transformer architectures have also emerged; SkinFormer embeds statistical texture descriptors into the transformer pipeline, achieving a reported Dice score of 93.2% [36]. Table 1 shows a summary of deep learning methods for lesion segmentation on the ISIC 2018 dataset.

Beyond segmentation, ISIC 2018 has also served as a benchmark for multi-class lesion classification, where performance has steadily improved through transfer learning, ensemble learning, and transformer-based representations. ESRGAN-enhanced transfer learning pipelines report accuracies near 85–86% on InceptionV3 baselines [37]. Multiscale and multi-network ensembles such as MSM-CNN achieve balanced accuracy of 86.2% [38]. Weighted transfer-learning ensembles further improve accuracy to 89.28% [39]. Lightweight transformer hybrids such as HI-MViT reach 93.2% accuracy, with 0.931 F1-score and AUC of 0.977 [40]. A three-network ensemble of VGG16, InceptionV3, and ResNet50 achieves 97% accuracy on a balanced version of ISIC 2018 [41]. Table 2 shows a summary of deep learning methods for lesion classification on the ISIC 2018 dataset.

Segmentation research on the PH2 dataset has similarly advanced. Lightweight CNN-MLP hybrids such as UCM-Net achieve Dice of 93% and IoU of 88.5% [42]. Semi-supervised DeepLabV3 teacher–student training yields mIoU of 87.54% [34]. Adversarial architectures such as EGAN report Dice above 92% [33]. Multiscale U-Nets such as MRP-UNet achieve Dice of 94.19% and IoU of 90.77% [35]. Deformable attention transformers further enhance boundary accuracy [43]. Table 3 shows a summary of deep learning methods for lesion segmentation on the PH2 dataset.

For the HAM10000 dataset, where only classification labels are available, transformer-based models such as SkinTrans achieve 94.3% accuracy [44]. Deep ensembles such as VGG16+InceptionV3+ResNet50 achieve 97% accuracy [41]. Hybrid CNN–capsule models such as Skin-DeepNet achieve precision and recall above 98.9% [45]. ViT variants such as ViT-HAM achieve strong binary and multi-class AUC performance [46]. ABC ensembles combining CNNs and transformers reach 95–96% accuracy [47]. Table 4 shows a summary of deep learning methods for lesion classification on the HAM10000 dataset.

Although recent advances have substantially improved skin lesion segmentation and classification, several important research gaps remain unaddressed. First, most segmentation models rely on task-specific CNN or transformer backbones trained from scratch or via conventional transfer learning, which limits their ability to generalize across diverse datasets and imaging domains; foundation-model-based segmentation has not yet been fully exploited in dermoscopy. Second, existing classification pipelines largely operate on full images or cropped regions without explicitly integrating segmentation priors, resulting in weak alignment between spatial lesion boundaries and semantic feature representations. Third, segmentation and classification are typically optimized as independent tasks, preventing mutual reinforcement and reducing robustness under dataset shift, class imbalance, or the presence of background artifacts such as hair, illumination variation, and marker noise. Finally, despite promising results on individual datasets, few works present unified architectures capable of achieving consistent performance across ISIC 2018, PH2, and HAM10000 in a harmonized and scalable manner.

While some newly published dermoscopic segmentation and classification research has been shown numerically to do well, further examination reveals that much of the improvement has been based on the underlying architecture of the model(s) used, and they are still limited by optimizations specific to the dataset(s) used in those studies. For instance, although many of the segmentation models that utilize either CNNs or hybrid CNN–Transformer architectures attain excellent Dice or IoU scores by virtue of improved methods for extracting features at multiple scales or employing multi-headed attention, the reliance on training data that are task specific hinders the ability for these models to generalize across domain shifts, which leads to low confidence in the predictions made when using independent datasets for external validation. Another example is that although transformer-based classifiers allow for better modeling of the global context of images, they do not provide any explicit awareness of lesion boundaries, and, therefore, predictions made from these models are considered to have a high risk of producing results that are heavily influenced by artifacts present in the background, such as hair, variations in lighting, or markers present from the acquisition process. A major limitation of current methods, including those mentioned above, is that most of the previously published approaches view the tasks of segmentation and classification as discrete from one another, rather than as complementary; therefore, there is no potential for mutual reinforcement between the spatial localization of a lesion and the semantic diagnosis of that lesion. Therefore, the limitations identified herein highlight the need for the development of a foundation-model-driven framework where the spatial location of a lesion, as indicated by segmentation, will be utilized as a prior when classifying that lesion, and, conversely, the identified diagnosis of the lesion would allow the segmentation of that lesion to be increased in accuracy across heterogeneous datasets when the previously mentioned joint optimization concept is utilized.

## 3. Proposed Methodology

**Figure 1:**
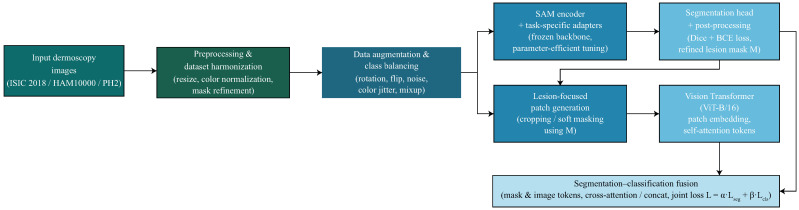
Overall SAM-ViT pipeline for dermoscopic skin lesion analysis.

The proposed framework introduces a foundation-model-driven pipeline for skin lesion segmentation and classification by integrating SAM-Adapter fine-tuning with ViT-based feature extraction. The methodology consists of nine components, forming a unified system that processes dermoscopic images, extracts lesion boundaries, learns discriminative representations, and generates clinically meaningful diagnostic outputs. An overview of the proposed foundation-model-driven SAM-ViT framework is shown in Figure 1.

### 3.1. Dataset Preparation and Harmonization

The preparation and harmonization of dermoscopic data constitute the foundational stage of the proposed framework, ensuring that all subsequent segmentation and classification operations occur on a consistent, normalized, and diagnostically meaningful representation of the input images. The datasets employed in this study—ISIC 2018, HAM10000, and PH2—originate from different acquisition devices, clinical environments, and annotation protocols, which naturally introduce substantial variability in spatial resolution, illumination properties, color distribution, and segmentation mask quality. In order to reduce these differences, we represent each input dermoscopy image as $I\in R^{H\times W\times 3}$, where *H* is the height and *W* is the width of the image, and the last dimension corresponds to the RGB color channels. The first step involved in the harmonization of our images is to rescale all images to a common resolution $\left(H_{0},W_{0}\right)$ with an interpolation-based resizing operator R(·) so that we are able to extract patches uniformly, and each function that is part of the subsequent foundation-model pipeline operates on an input image of the same resolution. Thus, the harmonized image $I^{*}$ is completed with the following:

(1) $$I^{*}=R\left(I\right),$$

where $R:R^{H\times W\times 3}\to R^{H_{0}\times W_{0}\times 3}$ ensures fixed spatial support. Despite standardization of resolution, dermoscopic images often exhibit significant differences in global illumination, camera white-balance settings, and pigmentation contrast, which hinder the stability of transformer-based feature extraction. To eliminate such inconsistencies, a histogram specification procedure is employed. Let $\hat{p}$ denote a reference intensity histogram computed as the median distribution across the training set. For each pixel location *x*, the normalized image $\tilde{I}$ is computed as

(2) $$\tilde{I}\left(x\right)=H\;\left(I^{*}\left(x\right),\hat{p}\right),$$

where H(·) represents the histogram-matching operator to match $I^{*}$ to the canonical distribution $\hat{p}$ to generate perceptually consistent images while retaining structural elements. Additionally, many images contain hair occlusions, shadows, vignetting artifacts, and disparate backgrounds and textures. Though our later segmentation module will mitigate some of these issues, it is important for our preprocessing to minimize the propagation of unwanted noise. When we have segmentation masks $M\in {\left\{0,1\right\}}^{H_{0}\times W_{0}}$, they are often biased in the annotation due to polygonal edges, over-segmentation, and isolated binary components. To correct these artifacts, we apply a morphological refinement operator $\phi_{\mathrm{morph}}\left(\cdot \right)$ consisting of dilation, erosion, and contour-smoothing filters to clean up the mask to obtain $M^{*}$ specified as

(3) $$M^{*}=\phi_{\mathrm{morph}}\left(M\right).$$

Although $M^{*}$ is a more coherent representation of the lesion boundary, smooth probabilistic boundaries are useful in transformer-based architectures that use continuous attention fields and do not rely on strict binary masks. Soft mask $M^{s}$ is created by smoothing $M^{*}$ with a Gaussian-blurred version which is denoted as $\psi (M^{*})$ with a smoothing coefficient α∈[0,1] such that

(4) $$M^{s}\left(x\right)=\alpha M^{*}\left(x\right)+\left(1-\alpha \right)\;\psi \left(M^{*}\right)\left(x\right).$$

This results in softer boundary zones that maintain anatomical accuracy while removing any artifacts of discretization. In the next stage, we will split each dataset into groups for training, validation, and testing on a per-patient basis so as to avoid leakage of identity. Let the full set of patient identifiers be $\left(P_{\mathrm{train}},P_{\mathrm{val}},P_{\mathrm{test}}\right)$, being mutually exclusive subsets of patient identifier space such that

(5) $$P_{\mathrm{train}}\cap P_{\mathrm{val}}=\emptyset ,\;P_{\mathrm{val}}\cap P_{\mathrm{test}}=\emptyset ,\;P_{\mathrm{train}}\cap P_{\mathrm{test}}=\emptyset .$$

This rigorous partition maintains that the same person’s image does not appear in more than one partition to avoid optimistic bias in classification accuracy. The final curated dataset splits are organized as follows:

(6) $$D_{k}=\left\{\left(\tilde{I},M^{s}\right)\;|\;\mathrm{patient}\left(I\right)\in P_{k}\right\},\;k\in \left\{\mathrm{train},\mathrm{val},\mathrm{test}\right\},$$

**Figure 2:**
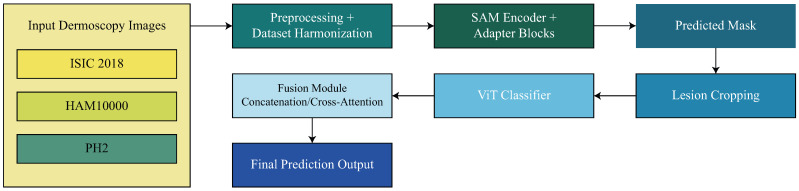
Overview of dataset preparation and harmonization, including unified preprocessing of ISIC 2018, HAM10000, and PH2 dermoscopic images.

This step ensures that every image–mask pairing is consistently aligned and fully harmonized with the pipeline’s preprocessing stage. While this degree of standardization also helps enhance the robustness of the SAM-Adapter segmentation phase of the proposed methodology, the benefit provided during ViT embedding extraction is perhaps the most significant, and is vital for the full strength of the proposed methodology. The dataset harmonization process is shown in Figure 2.

### 3.2. Data Augmentation and Class Balancing

The variability across dermoscopic datasets often presents itself not only through imaging conditions, but also substantial imbalance amongst diagnostic categories, especially concerning malignant lesions (e.g., melanoma), as they represent a minority class relative to benign nevi. To help stabilize and avoid bias throughout the learning process across all lesion categories, a comprehensive data augmentation and balancing approach is introduced. Denote each input dermoscopy image by $I\in R^{H_{0}\times W_{0}\times 3}$, and let A(·) represent a stochastic augmentation operation that invokes a sequence of transformations to increase invariance to pose, illumination, and acquisition. The augmented sample $\tilde{I}$ is now defined as

(7) $$\tilde{I}=A\left(I\right),$$

where A(·) encompasses random rotations, horizontal and vertical flips, elastic deformations, random affine transformations, Gaussian noise injection, color jitter, and brightness–contrast perturbations. More formally, let $T_{r}$ denote a random rotation by an angle θ drawn from a finite interval Θ, let $T_{f}$ denote flipping along the spatial axes, and let $T_{n}$ denote pixel-wise noise injection. Then, the augmentation pipeline can be expressed as a compositional map

(8) $$A\left(I\right)=T_{n}\left(T_{f}\left(T_{r}\left(I\right)\right)\right),$$

highlighting that the modifications are made in a sequential, probabilistic manner, in order to augment structural and chromatic variability. Aside from this, affine transformations are especially useful for improving robustness to geometric distortions. An affine-transformed image $I_{\mathrm{aff}}$ is obtained using the mapping $x^{\prime }=Ax+b$, with $A\in R^{2\times 2}$ and $b\in R^{2}$, as

(9) $$I_{\mathrm{aff}}\left(x^{\prime }\right)=I\left(A^{-1}\left(x^{\prime }-b\right)\right),$$

where *A* is sampled from a distribution of small perturbations around the identity matrix, ensuring realistic deformations without excessive distortion of lesion boundaries. Color jittering is applied to increase diversity in pigmentation patterns and is modeled as a transformation on the intensity vector I(x) at pixel *x* such that

(10) $$I_{\mathrm{jitter}}\left(x\right)=\gamma \;I\left(x\right)+\delta ,$$

where γ and δ are randomly sampled gain and bias values chosen to simulate variations in illumination and sensor response. While traditional augmentation enhances generalization, severe class imbalance—typical in dermatology datasets—requires explicit balancing strategies. Let C={1,2,…,C} denote the set of lesion classes and $N_{c}$ denote the number of samples belonging to class c∈C. When the distribution is highly skewed, synthetic augmentation such as mixup is applied, producing mixed samples $\tilde{I}$ and corresponding mixed labels $\tilde{y}$ as

(11) $$\tilde{I}=\lambda I_{a}+\left(1-\lambda \right)I_{b},\;\tilde{y}=\lambda y_{a}+\left(1-\lambda \right)y_{b},$$

where $I_{a}$ and $I_{b}$ denote two randomly sampled images, $y_{a},y_{b}\in {\left\{0,1\right\}}^{C}$ are their one-hot encoded class labels, and λ is drawn from a Beta distribution

**Figure 3:**
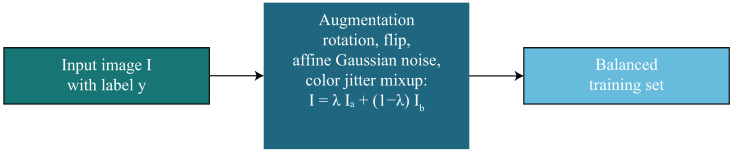
Data augmentation and class balancing pipeline, including geometric transformations, photometric perturbations, and mixup-based rebalancing.

(12) λ∼Beta(α,α),

with α>0 controlling the strength of interpolation. Furthermore, to promote balanced sampling across classes, we introduce a class-weighting scheme where the probability of drawing a sample from class *c* is inversely proportional to its frequency, defined as

(13) $$p\left(c\right)=\frac{1/N_{c}}{\sum_{k=1}^{C}\left(1/N_{k}\right)},$$

ensuring that rare classes such as melanoma are sampled more frequently during training. In addition, we incorporate focal reweighting to prevent domination of the loss by well-represented classes. For an image–label pair (I,y), the class-balanced weight $w_{c}$ for category *c* is expressed as

(14) $$w_{c}=\frac{1-\beta }{1-\beta^{N_{c}}},$$

where β∈[0,1) controls the degree of balancing, with larger values placing greater emphasis on minority categories. The combination of geometric and photometric augmentations, mixup-based synthetic generation, inverse-frequency sampling, and class-dependent loss reweighting establishes a robust and balanced dataset foundation, enabling the segmentation and classification modules to learn invariant, discriminative, and clinically meaningful features while mitigating the detrimental effects of class imbalance. The augmentation and balancing strategy is summarized in Figure 3.

### 3.3. SAM-Adapter Fine-Tuning

The SAM serves as the foundational segmentation backbone due to its extensive pretraining on billions of masks and its strong generalization capabilities across diverse visual domains. However, directly fine-tuning the entire SAM encoder for a highly specialized dermoscopic segmentation task is computationally expensive and may lead to catastrophic forgetting of its general-purpose representations. To avoid these drawbacks, we adopt a parameter-efficient fine-tuning strategy in which lightweight adapter modules are inserted into the encoder blocks.

When embedding SAM capabilities from an original foundation into images taken with a dermoscopic, creating a separate adapter module allows for several technical advantages that will be covered in this section. First, while the original SAM was designed and tested to complete multiple tasks based on natural images, creating new model types with this same capability will require significantly more computing resources and would most likely result in removing all of the pretrained weights. Second, by using an adapter module approach, we will reduce the number of trainable parameters needed within a single SAM model and refine the original model’s features without removing any of the accurate representation of the backbone network. Third, through this modular approach, we will have an improved set of SAM features, resulting in increased flexibility and training stability for training with small sets of medical imaging data, which can potentially lead to more effective use cases for adapting dermoscopic lesions into the SAM architecture. Fourth, and as demonstrated through the results of our ablation experiments, when using an adapter module to refine the features generated by the original frozen SAM model versus only using the frozen SAM model, the results produced will provide an increased level of segmentation accuracy, thereby supporting the use of an adapter module when transitioning from zero-shot segmentation to fine-tuning of a single SAM model.

Let the original SAM image encoder be denoted by $E_{\mathrm{SAM}}:R^{H_{0}\times W_{0}\times 3}\to R^{d\times h\times w}$, where *d* represents the hidden feature dimension and (h,w) denote the downsampled spatial dimensions produced by the hierarchical ViT architecture. For an input harmonized image $I^{*}$, the raw SAM feature map is

(15) $$Z=E_{\mathrm{SAM}}\left(I^{*}\right),$$

which encodes multiscale contextual information. To adapt these features to dermoscopic lesion boundaries without modifying the large backbone, a learnable adapter module $A_{\theta }$ with parameters θ is introduced. Conceptually, the adapter applies a low-rank transformation or bottleneck mapping to the encoded representation. The adapted feature map *F* is thus defined as

(16) $$F=Z+A_{\theta }\left(Z\right),$$

where the additive residual structure ensures that the pretrained SAM features are preserved while task-specific refinements are introduced. A common design for $A_{\theta }$ uses a down-projection and up-projection structure, often referred to as a bottleneck adapter. In the proposed SAM-Adapter design, the adapter modules are integrated directly within the transformer blocks of the SAM image encoder. Specifically, for each transformer layer of the SAM encoder, the adapter is inserted after the feed forward network (FFN) sublayer and operates in a residual manner on the layer output. Let $Z^{\left(𝓁\right)}$ denote the output of the *ℓ*-th transformer block in the frozen SAM encoder after self-attention and FFN operations. The adapted representation is computed as

(17) $$F^{\left(𝓁\right)}=Z^{\left(𝓁\right)}+A_{\theta }\;\left(Z^{\left(𝓁\right)}\right),$$

where $A_{\theta }\left(\cdot \right)$ denotes the lightweight bottleneck adapter with learnable parameters θ. This placement allows the adapter to refine high-level contextual representations learned by SAM while preserving the pretrained attention structure and global segmentation priors. Adapter modules are applied uniformly across all transformer layers of the SAM image encoder, whereas the prompt encoder and mask decoder components of SAM remain unchanged and fully frozen during training. Let $W_{↓}\in R^{d\times r}$ and $W_{↑}\in R^{r\times d}$ denote learnable projection matrices, with r≪d representing the adapter rank. Then, the adapter transformation takes the form

(18) $$A_{\theta }\left(Z\right)=W_{↑}\;\sigma \;\left(W_{↓}Z\right),$$

where σ(·) is a point-wise nonlinearity such as GELU, and $\theta =\{W_{↓},W_{↑}\}$. This low-rank decomposition allows to dramatically reduce trainable parameters and stay computationally feasible. A LoRA-based adapter can also be used, where the weight update is performed in a low-rank manner to the attention projection matrices. A pretrained matrix $W_{0}$ in the SAM encoder has a LoRA-modified version of

(19) $$W=W_{0}+BA,$$

where $A\in R^{r\times d}$ and $B\in R^{d\times r}$ are learnable low-rank matrices. This modification to the SAM that is efficient in parameters preserves the SAM global representational power, and enables extremely specific adaptation to medical segmentation. To enhance representation, we propose a gated adapter mechanism to modulate the contribution of adapter by an input dependent gate g(Z) shown as:

(20) $$g\left(Z\right)=\sigma \;\left(W_{g}Z+b_{g}\right),$$

with $W_{g}$ and $b_{g}$ being learnable parameters. The final gated-adapter output is then expressed as

(21) $$F=Z+g\left(Z\right)⊙A_{\theta }\left(Z\right),$$

where ⊙ refers to element-wise multiplication. This approach enables network adaptively alters the influence of the adapter based on the complexity of the lesion, texture variability or color variation. During training, only the adapter parameters θ (and optionally the gating parameters) are optimized while the entire SAM backbone remains frozen. If $L_{\mathrm{seg}}$ denotes the segmentation loss combining Dice and binary cross-entropy terms, the optimization process is formally written as

(22) $$\theta^{*}=\arg\min_{\theta }\;L_{\mathrm{seg}}\;\left(M,\hat{M}\left(F\right)\right),$$

where *M* is the ground-truth mask and $\hat{M}\left(F\right)$ is the predicted segmentation mask produced by the SAM decoder using the adapted features. By restricting optimization to a small parameter subset, the SAM-Adapter mechanism not only maintains the powerful pretraining priors of the original model but also enables effective specialization to the fine-grained lesion boundary detection required in dermoscopic images. This efficient adaptation forms the basis upon which segmentation-guided lesion extraction and subsequent ViT-based classification are built, ensuring coherent information flow throughout the entire proposed framework. The SAM-Adapter fine-tuning strategy is depicted in Figure 4.

### 3.4. Segmentation-Guided Lesion Extraction

Following SAM-Adapter fine-tuning, the model produces a pixel-wise probability map that encodes the likelihood of each pixel belonging to a lesion. Let this probability map be denoted by p:Ω→[0,1], where $\Omega =\left\{1,\ldots ,H_{0}\right\}\times \left\{1,\ldots ,W_{0}\right\}$ represents the spatial domain of the harmonized image $I^{*}$. Each value p(x) corresponds to the model’s confidence that pixel *x* belongs to the lesion region. To derive a binary segmentation mask suitable for downstream processing, a thresholding operator $T_{\tau }\left(\cdot \right)$ is applied using a threshold τ∈[0,1], yielding the initial predicted mask $\hat{M}$ as

(23) $$\hat{M}\left(x\right)=⊮\left(p(x)\geq \tau \right),$$

where ⊮(·) denotes the indicator function that outputs 1 if the condition inside is satisfied and 0 otherwise. However, since dermoscopic lesion boundaries often exhibit irregular textures, soft edges, and artifacts such as hair, shadows, and illumination gradients, the raw mask $\hat{M}$ may contain spurious noise, small disconnected components, or jagged boundaries. To correct these imperfections, a contour-smoothing operator $\phi_{\mathrm{smooth}}\left(\cdot \right)$ is applied to generate a refined mask ${\hat{M}}^{*}$. This operator may include morphological closing, contour regularization, and Gaussian boundary smoothing, expressed formally as

(24) $${\hat{M}}^{*}=\phi_{\mathrm{smooth}}\left(\hat{M}\right),$$

where $\phi_{\mathrm{smooth}}:{\left\{0,1\right\}}^{H_{0}\times W_{0}}\to {\left[0,1\right]}^{H_{0}\times W_{0}}$ yields a soft boundary representation that represents the continuous anatomical structure of dermoscopic lesions. To reduce local inconsistencies and to reduce isolated false positives, we also include a connected-component filtering operator C(·) which keeps only the largest connected part of the lesion. If $L({\hat{M}}^{*})$ is the set of all connected components, we update the final mask as

(25) $${\hat{M}}^{†}\left(x\right)=\left\{\begin{array}{cc} {\hat{M}}^{*}\left(x\right), & \mathrm{if}\;x\in \arg\max_{C\in L({\hat{M}}^{*})}\left|C\right|,\\ 0, & \mathrm{otherwise}, \end{array}\right.$$

ensuring that only the dominant lesion structure is preserved for subsequent analysis. Once the segmentation mask is refined, it is used to extract the lesion-focused region of interest. Let ⊙ denote element-wise multiplication between an image and a mask (broadcast over channels). The lesion-specific image representation $I_{\mathrm{les}}$ is then obtained as

(26) $$I_{\mathrm{les}}=I^{*}⊙{\hat{M}}^{†}.$$

**Figure 5:**
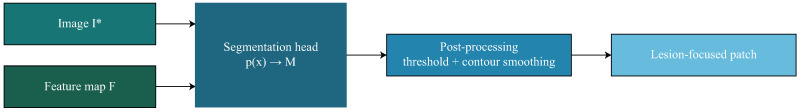
Segmentation-guided lesion extraction pipeline. Predicted masks undergo thresholding, contour smoothing, and cropping to produce lesion-focused patches.

This operation suppresses background tissue, artifacts, and irrelevant regions, retaining only the lesion pixels while preserving original color and texture information. Furthermore, for tasks requiring explicit lesion cropping, a bounding box $B=(x_{\min},x_{\max},y_{\min},y_{\max})$ is derived by computing the minimum rectangle enclosing all pixels where ${\hat{M}}^{†}\left(x\right)>0$. The cropped lesion patch is then obtained as

(27) $$I_{\mathrm{crop}}=I^{*}\left[x_{\min}:x_{\max},\;y_{\min}:y_{\max}\right],$$

which focuses the classification model on the most diagnostically relevant region. To maintain spatial consistency with later fusion stages, a softly weighted lesion representation is also constructed by blending the full image with the segmentation mask:

(28) $$I_{\mathrm{soft}}\left(x\right)=\beta I^{*}\left(x\right){\hat{M}}^{†}\left(x\right)+\left(1-\beta \right)I^{*}\left(x\right),$$

where β∈[0,1] controls the strength of lesion emphasis. This formulation ensures that the classifier receives lesions with enhanced contrast while preserving contextual cues in the surrounding tissue. The combination of thresholding, morphological refinement, connected-component filtering, cropping, and soft foreground weighting forms a robust mechanism that isolates the lesion region, thereby providing a clean, semantically focused input for the ViT feature extraction stage that follows in the pipeline. The segmentation-driven lesion extraction process is shown in Figure 5.

### 3.5. Vision Transformer Feature Extraction

While convolutional neural networks have been shown to perform well at analyzing dermoscopic images, they are limited by their reliance on local receptive fields and hierarchical aggregation of features. Therefore, these neural networks are less capable of building long-range contextual dependencies across the entire lesion, especially when compared with Vision Transformers. Vision Transformers analyze a whole image and allow for direct relationships between far-apart regions in an image, which makes them advantageous for analyzing global characteristics of lesions. Additionally, Transformer token representations present a natural way to integrate segmented spatial priors, whereby cross attention integrates the image features with lesion segmentation at the token level. Conversely, CNN pipelines have difficulty fusing different modalities as the dense nature of feature maps makes them less adaptable to varied ways of integration. Given these advantages, Vision Transformers are chosen as the basis for the classification task, as they provide efficient lesion-centric reasoning and facilitate accurate integration of segmentation and classification while leveraging the power of pretrained representations for reliable generalization.

Once a lesion-focused representation has been generated through segmentation-guided extraction, the next stage of the pipeline employs a ViT to learn rich, non-local, and semantically discriminative features from either the full dermoscopic image or the masked lesion region. Let the selected input image be denoted by $I_{\mathrm{in}}\in R^{H_{0}\times W_{0}\times 3}$, where $I_{\mathrm{in}}$ may correspond to $I^{*}$, $I_{\mathrm{les}}$, or $I_{\mathrm{soft}}$, depending on the fusion strategy. The ViT architecture operates by decomposing the spatial domain $\Omega =\left\{1,\ldots ,H_{0}\right\}\times \left\{1,\ldots ,W_{0}\right\}$ into a collection of non-overlapping square patches of fixed resolution p×p. Let the total number of patches be $N=\frac{H_{0}W_{0}}{p^{2}}$. Each patch $P_{i}$ is extracted by slicing the input image at coordinates $\left(x_{i},y_{i}\right)$ according to

(29) $$P_{i}=I_{\mathrm{in}}\left[x_{i}:x_{i}+p,\;y_{i}:y_{i}+p\right],$$

ensuring consistent partitioning across the entire image grid. To map each raw patch into a latent feature space, the 2D array is vectorized using vec(·) and then transformed via a trainable linear projection matrix $W_{\mathrm{proj}}\in R^{d\times (3p^{2})}$, where *d* is the transformer embedding dimension. The patch embedding $z_{i}$ is therefore computed as

(30) $$z_{i}=W_{\mathrm{proj}}\;\mathrm{vec}\left(P_{i}\right),$$

resulting in a series of *N* patch tokens. Since transformers are mode independently permuted, positional encodings are added to maintain spatial locality. Let $e_{i}\in R^{d}$ denote the positional vector corresponding to the location of patch *i*. A learnable class token $z_{\mathrm{cls}}\in R^{d}$ is prepended to the sequence to aggregate global lesion semantics during forward propagation. Thus, the sequence of tokens $Z_{0}$ passed into the first transformer layer can be written as

(31) $$Z_{0}=\left[z_{\mathrm{cls}},\;z_{1}+e_{1},\;z_{2}+e_{2},\;\ldots ,\;z_{N}+e_{N}\right],$$

creating a structured format that combines both local texture cues from patch embeddings and global spatial context with positional encodings. In each transformer layer *ℓ*, multi-head self-attention is performed to capture long-range dependencies across different regions of an image, such as pigment networks, streaks, globules, and border irregularities. Let $Z_{𝓁-1}$ denote the input token matrix at layer *ℓ*. The layer first computes query, key, and value projections using learnable matrices $W_{Q}^{\left(𝓁\right)},W_{K}^{\left(𝓁\right)},W_{V}^{\left(𝓁\right)}$. Specifically,

(32) $$Q=Z_{𝓁-1}W_{Q}^{\left(𝓁\right)},\;K=Z_{𝓁-1}W_{K}^{\left(𝓁\right)},\;V=Z_{𝓁-1}W_{V}^{\left(𝓁\right)}.$$

**Figure 6:**
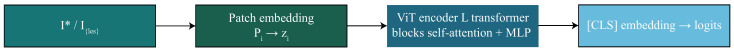
ViT-based feature extraction pipeline. Input patches are embedded, processed through transformer blocks, and summarized via the [CLS] token.

Self-attention is then computed across all token pairs. For each head, the attention map is derived using scaled dot-product attention, defined as

(33) $$\mathrm{Attention}\left(Q,K,V\right)=\mathrm{softmax}\;\left(\frac{QK^{⊤}}{\sqrt{d}}\right)V,$$

where the dimensionality of each of the embeddings is *d*, and the scaling term $\frac{1}{\sqrt{d}}$ is used to stabilize the magnitudes of the gradients. Each attention head (there are *h* total heads) produces an output that we concatenate before performing a linear projection to get the output attention $A_{𝓁}$. We then add a residual connection and apply a feed forward network (FFN). Specifically, layer 𝓁−1 to layer *ℓ* is given by

(34) $${\tilde{Z}}_{𝓁}=Z_{𝓁-1}+A_{𝓁}\left(Z_{𝓁-1}\right),$$

(35) $$Z_{𝓁}={\tilde{Z}}_{𝓁}+\mathrm{FFN}\left({\tilde{Z}}_{𝓁}\right),$$

where FFN(·) is composed of two linear layers combined with an activation function, such as GELU. Stack layers help the model to absorb global information across distant areas of the lesion and recognize irregularities such as asymmetry, border differences, or heterogeneous pigmentation. The output in the final layer for the class token $z_{\mathrm{cls}}^{\left(L\right)}$ summarizes the complete representation of the lesion image. This class token is used in the classification head’s decision-making for malignancy prediction. The ViT learns a powerful hierarchical representation from a patch decomposition, token embedding, positional encoding, and subsequent multi-layer self-attention, and this representation will support the fusion-based decision-making at later stages of the proposed framework. The ViT feature extraction flow is shown in Figure 6.

### 3.6. Segmentation–Classification Fusion

A primary goal of the proposed framework is to create a tight coupling between the spatial priors from segmentation and the transformer-based semantic representations, to allow the classifier to attend to clinically relevant lesion shapes but also retain context cues which are essential for reliable decision-making. To achieve this integration, a dedicated fusion module is introduced that combines two distinct forms of information: (i) image embeddings derived from the ViT feature extractor, and (ii) mask-driven embeddings produced from the segmentation maps generated by the SAM-Adapter. Let $Z_{\mathrm{img}}\in R^{(N+1)\times d}$ denote the sequence of embeddings obtained from the ViT encoder, where *N* is the number of image patches, and the extra token corresponds to the class token. Similarly, let $Z_{\mathrm{mask}}\in R^{(N+1)\times d}$ denote the mask embeddings, constructed by flattening the refined mask ${\hat{M}}^{†}$ into patch-level representations and projecting them using a learnable mask-embedding matrix $W_{\mathrm{mask}}\in R^{d\times p^{2}}$. Formally, if $M_{i}$ denotes the mask patch corresponding to image patch $P_{i}$, then the associated embedding is

(36) $$m_{i}=W_{\mathrm{mask}}\;\mathrm{vec}\left(M_{i}\right),$$

and the full mask embedding sequence is $Z_{\mathrm{mask}}=\left[m_{\mathrm{cls}},m_{1},\ldots ,m_{N}\right]$, where $m_{\mathrm{cls}}$ is a learnable token encoding global lesion shape prior. The goal of the fusion module is to integrate these two embedding streams into a joint representation $Z_{\mathrm{fusion}}$ that carries both lesion-localized spatial constraints and global semantic descriptors. A simple yet effective fusion technique is concatenation along the embedding dimension, which preserves the identity of both streams while allowing the downstream classifier to learn optimal weighting. This fusion operation is defined as

(37) $$Z_{\mathrm{fusion}}=\left[Z_{\mathrm{img}}\;∥\;Z_{\mathrm{mask}}\right],$$

where ∥ denotes concatenation along the feature axis. Although concatenation preserves full information, it does not explicitly model cross-stream interactions. To mitigate this drawback, we introduce a cross-attention-based fusion mechanism that allows the image embeddings to directly attend to cues from the mask-derived features and vice versa. Let the query matrix be obtained from the image embeddings as follows:

(38) $$Q_{\mathrm{img}}=Z_{\mathrm{img}}W_{Q}^{\mathrm{img}},$$

and the key and value matrices derived from the mask embeddings as

(39) $$K_{\mathrm{mask}}=Z_{\mathrm{mask}}W_{K}^{\mathrm{mask}},\;V_{\mathrm{mask}}=Z_{\mathrm{mask}}W_{V}^{\mathrm{mask}},$$

where $W_{Q}^{\mathrm{img}},W_{K}^{\mathrm{mask}},W_{V}^{\mathrm{mask}}\in R^{d\times d}$ are learned projection matrices. Cross-attention fusion is then computed using the scaled dot-product formulation

(40) $$Z_{\mathrm{fusion}}=\mathrm{softmax}\;\left(\frac{Q_{\mathrm{img}}K_{\mathrm{mask}}^{⊤}}{\sqrt{d}}\right)V_{\mathrm{mask}}.$$

**Figure 7:**
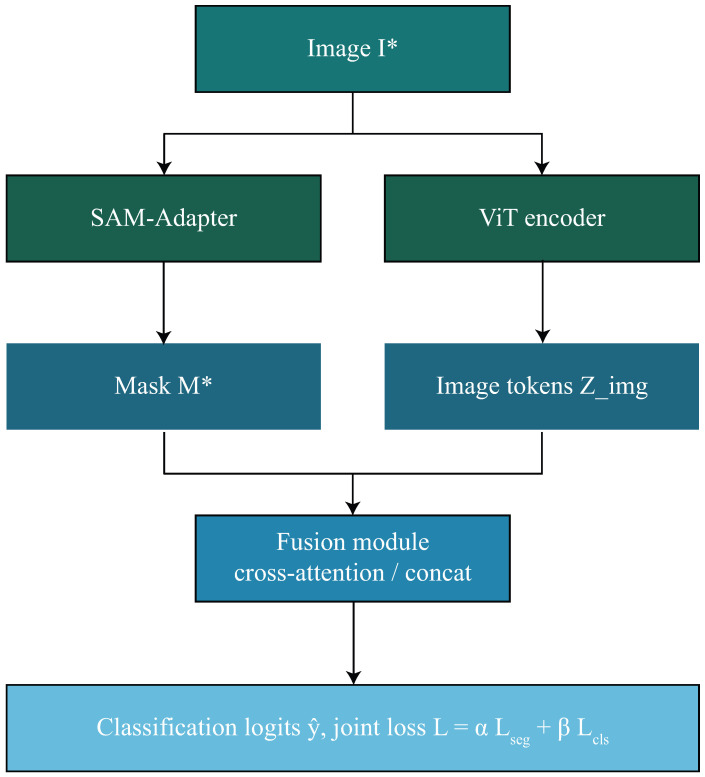
Joint segmentation–classification architecture. SAM-derived mask embeddings and ViT image embeddings are integrated via concatenation or cross-attention fusion.

This approach makes sure that every image token selectively attends to the mask regions that are most informative of lesion pathology, combining the structure of lesion boundaries with the interpretation of the semantic features altogether. To improve the adaptivity of the feature fusion, a gating mechanism is proposed that allows the model to dynamically control how much the segmentation contributes to the fused representation. We denote $g\in {\left[0,1\right]}^{(N+1)\times 1}$ as the learnable gating vector resulting from

(41) $$g=\sigma \;\left(Z_{\mathrm{img}}W_{g}+b_{g}\right),$$

where $W_{g}\in R^{d\times 1}$ and $b_{g}\in R^{1}$ are learnable parameters. The final gated fusion representation is therefore expressed as

(42) $$Z_{\mathrm{fusion}}^{†}=g⊙Z_{\mathrm{fusion}}+\left(1-g\right)⊙Z_{\mathrm{img}},$$

where ⊙ denotes element-wise multiplication across tokens. This formulation permits the model to attend to segmentation cues when the lesion boundaries are sharp and reliable, while enabling the sequential multiplication to optimally rely on the derived semantics when the segmentation is uncertain or noisy. Utilizing a combination of concatenation, cross-attention, and gating to represent the proposed fusion module as a unified bridge between low-level lesion structure and high-level semantic representation enables the downstream classification head to operate within a highly contextualized feature space. This finely tuned representation materially improves the model’s capacity to separate subtle malignancy cues in complex dermoscopic images. Most importantly for the next joint optimization component, it provides the basis for the model’s segmentation-classification fusion architecture in Figure 7.

### 3.7. Joint Optimization

The framework is built around a unified joint optimization approach by tightly coupling segmentation-driven spatial priors obtained from the SAM-Adapter, with high-level learned semantic descriptors from the ViT. We do this joint optimization in two phases that are distinct, yet related, namely segmentation training and classification training. Together, this will allow the model to capture lesion morphology, texture, boundary irregularities, and multi-class diagnostic cues to perform robust skin cancer analysis. The training format is developed so that segmentation aids classification by improving the localization of lesions, while classification gradients ensure the network does prioritize discriminative lesion features. This mutually reinforcing approach is key to developing a consistent end-to-end learning framework that is reliable, even when datasets have high levels of heterogeneity in class distribution, image appearance, and/or granularity of annotation.

In the first phase of the training, segmentation training, we aim the model to learn precise lesion boundaries to inform classification in the next phase. Let M(x)∈{0,1} be the ground-truth mask and $\hat{M}\left(x\right)\in \left[0,1\right]$ the predicted segmentation probability at pixel *x*. The segmentation loss function $L_{\mathrm{seg}}$ is defined as a combination of Dice loss and binary cross-entropy (BCE) loss, which measures performance at the region level and consistency in pixel-wise probability respectively. That is,

(43) $$L_{\mathrm{seg}}=L_{\mathrm{Dice}}+L_{\mathrm{BCE}},$$

where the Dice loss penalizes overlap mismatch and is defined as

(44) $$L_{\mathrm{Dice}}=1-\frac{2\sum_{x\in \Omega }\hat{M}\left(x\right)\;M\left(x\right)}{\sum_{x\in \Omega }\hat{M}\left(x\right)+\sum_{x\in \Omega }M\left(x\right)},$$

with Ω representing the spatial pixel grid. To complement this, the BCE term accounts for per-pixel classification fidelity and is expressed as

(45) $$L_{\mathrm{BCE}}=-\sum_{x\in \Omega }\left(M\left(x\right)\log\hat{M}\left(x\right)+\left(1-M\left(x\right)\right)\log\left(1-\hat{M}\left(x\right)\right)\right).$$

During this phase, only the adapter parameters θ embedded within the SAM-Adapter are updated, while the rest of the SAM encoder remains frozen. The optimization objective for segmentation is therefore

(46) $$\theta^{*}=\arg\min_{\theta }\;L_{\mathrm{seg}}\left(M,\hat{M}\right),$$

ensuring efficient and domain-specific adaptation without overfitting, or eroding the pretrained representational strength of SAM. In the second phase, the classification training leverages refined lesion-focused images and fused embeddings to learn high-level diagnostic distinctions. Let $y=(y_{1},\ldots ,y_{C})$ denote the one-hot vector representing the ground-truth class label among *C* lesion categories, and let ${\hat{y}}_{c}$ denote the predicted class probability for class *c*. The classification loss $L_{\mathrm{cls}}$ is computed using the standard categorical cross-entropy:

(47) $$L_{\mathrm{cls}}=-\sum_{c=1}^{C}y_{c}\log\left({\hat{y}}_{c}\right),$$

which encourages the classifier to assign high probability to the correct lesion category while minimizing misclassification errors. To further enhance robustness in imbalanced datasets, class-dependent weights $w_{c}$ may be integrated, yielding the weighted cross-entropy

(48) $$L_{\mathrm{wcls}}=-\sum_{c=1}^{C}w_{c}y_{c}\log\left({\hat{y}}_{c}\right),$$

where $w_{c}=\frac{1}{N_{c}}$ or $w_{c}=\frac{1-\beta }{1-\beta^{N_{c}}}$ depends on the dataset properties, and $N_{c}$ is the number of samples in class *c*. In order to jointly account for the segmentation and classification goals (if desired), a joint loss, labeled $L_{\mathrm{joint}}$, can be applied during the fine-tuning step with balancing weights $\lambda_{\mathrm{seg}}$ and $\lambda_{\mathrm{cls}}$:

(49) $$L_{\mathrm{joint}}=\lambda_{\mathrm{seg}}L_{\mathrm{seg}}+\lambda_{\mathrm{cls}}L_{\mathrm{cls}},$$

with $\lambda_{\mathrm{seg}},\lambda_{\mathrm{cls}}>0$ dictating the influence of each term. In this way, the network aligns segmentation accuracy with classifying accuracy and thus ensures both morphological and semantic consistency. In total, these two training procedures dictate a coherent optimization path that draws on segmentation to refine the localization of features and relevant classification to supplement this with semantic abstraction, in order to create a fully complementary model capable of reliable and interpretable skin lesion analysis.

Nevertheless, although the development of SAM for medical image segmentation is still at the early stage in the literature, most of the existing methods use SAM either implicitly or apply some sort of fine-tuning, either partial or full, for performing just one single downstream task. Compared with this, the presented framework incorporates a parameter-efficiency adapter design, which is carefully integrated into each of the transformer blocks of the SAM image encoder, which allows domain-specific adaptation, while safeguarding the full pretrained attention structure and general-purpose segmentation priors. Such a design diverges from the previous forms of fine-tuning of SAM by explicitly constraining the capacity of trainable parameters and by enabling fine-grained lesion boundary changes, which are unrelated to generic mask matching.

In addition, unlike the existing guiding states for classification that rely mainly on hard lesion cropping or post hoc endorsements of masks, the method presented in this proposal has introduced a token-level fusion mechanism, whereby the SAM-of-lesion mask embeddings are directly incorporated with the Vision Transformer image tokens through cross-attention. This fusion allows the introduction of dynamic modulation of the semantic classification features conditioned on lesion-specific spatial priors and therefore treats the segmentation step rather as a prior active domain dissection layer than static preprocessing. This means that the lesion shape and boundary-related information further influence the whole diagnostic rationale looping inside the transformer layers themselves.

Finally, unlike traditional sequential training paradigms, in this study we look at the joint optimization scheme that distinctly couples the segmentation and classification objectives into a common space. On the other hand, most prior works rather occur independently, and the joint optimization parameterization gathers spatial cues obtained from segmentation to direct learning in classification, while supervision in classification provides feedback towards the fine-tuning of lesion features. Such bidirectionality acts therefore as a novel architectural contribution: not merely a mark of improvement but instead one that propagates the idea of coherent lesion-centric representation learning through heterogeneous data pools.

## 4. Experimental Results and Discussion

### 4.1. Datasets and Experimental Setup

Three publicly available dermoscopic datasets were used for the experiments: ISIC 2018, HAM10000, and PH2. Each dataset comprises distinct imaging conditions, lesion categories, and characteristics of annotation. The ISIC 2018 dataset includes pixel-level segmentation masks and seven-class diagnostic labels, allowing for evaluation of the entire segmentation–classification pipeline. Segmentation was performed on all 2594 training images of the corresponding classification subset, which had a total of 10,015 images, to evaluate lesion recognition. The HAM10000 dataset contains 10,015 dermoscopic images across seven diagnostic categories and does not contain segmentation masks. Thus, the HAM10000 dataset is aligned towards assessing the classification module across a range of possible real-world acquisition environments. The PH2 dataset contains 200 high-resolution dermoscopic images and associated precise ground truth manual lesion masks. Therefore, this dataset can be used to assess the segmentation quality for a highly manually curated dataset. All datasets were split using patient–disjoint splits to avoid identity leakage, where a 70% split was selected for training, 10% for validation, and 20% for testing unless otherwise specified.

In order to establish consistency among the various datasets, each image was resized to 224×224 and normalized by channel with respect to the ImageNet dataset statistics. Segmentation masks were available for some datasets; thus, morphology filtering and contour refinement were carried out on segmentation masks to remove annotation noise and smooth the masks. Data augmentation for each dataset made use of random horizontal and vertical flipping, $90^{\circ }$ rotations, affine transformations, Gaussian noise injection, and color jitter to brightness, contrast, and saturation. For the classification problem, the imbalance of the classes was addressed through a combination of weighted cross-entropy loss and mixup augmentation with a mixing coefficient λ∼Beta(α,α).

The proposed framework was implemented in PyTorch (version 2.1) and trained using an NVIDIA RTX 5070 GPU. The SAM encoder was kept frozen, and only the adapter parameters were optimized. The ViT backbone was initialized from ImageNet-pretrained weights and fine-tuned end-to-end. The AdamW optimizer was employed for both branches of segmentation and classification; an initial learning rate of $1\times 10^{-4}$, cosine learning rate decay, and a weight decay of $1\times 10^{-2}$ were utilized. The segmentation branch was optimized with a hybrid loss of Dice loss and binary cross-entropy, while the normal cross-entropy was employed for the classification branch. At the joint optimization phase, the overall loss function was treated as the weighted summation of segmentation and classification loss, where the weights were determined semi-empirically in an attempt to stabilize multi-task convergence.

Performance was evaluated using standard metrics. For the segmentation task, the following metrics were reported: the Dice coefficient; Jaccard index (IoU); sensitivity; specificity; and mean pixel accuracy. For the classification task, we report accuracy, precision, recall, F1-score, and area under the ROC curve (AUC). Each experiment was performed three times, with different random seeds, and mean performance values are reported for statistical significance. Throughout the process of using baseline comparisons, the train–validation–test splits were carried out in the same way to maintain fairness. The entire experimental process was aligned with the evaluation protocols for dermoscopic image analysis in the literature, maintaining both reproducibility and comparability with previous state-of-the-art methods.

The HAM10000 dataset has a significant class imbalance but many techniques were implemented to minimize overfitting and to achieve the validation of generalization from the data in training. First, all experiments were conducted using a patient–disjoint train–validation–test split; hence, all data used to validate the classifier were gathered from different patients than those used for training would be. Second, during training, a large number of augmentations created a more diverse set of samples to be used for training and allowed the classifier to have less chance of memorizing the dominant classes. Third, to address the class imbalance problem of the HAM10000 data, cross-entropy loss functions based on the respective classes were employed which allowed the classifier to apply a heavier penalty in misclassifications of minority classes compared with majority classes. Fourth, in addition to using a frozen foundation model as a segmentation backbone, only lightweight adapter modules were trained on the HAM10000 data; therefore, there was considerable reduction in model capacity, which decreases the risk of overfitting. Finally, as all results are represented as the average output from three independent trials using different random-seed initializations, this provides an additional validation of both the stability of the training process and the robustness of the classifier.

It should be stated clearly that the HAM10000 dataset does not contain true pixel-level ground-truth segmentation and is, therefore, only being used for classifying purposes in the proposed architecture as outlined above. Therefore, we do not report any quantitative evaluation of the segmentation metrics associated with HAM10000. Under this assumption, we use the SAM-Adapter segmentation branch solely for the purpose of generating image-level representations focused on, or directed toward, the classification task and to provide additional localizing support to the Vision Transformer. The SAM-Adapter segmentation branch was trained and quantitatively evaluated exclusively on datasets for which ground-truth segmentation masks have been made available, but the outputs of the SAM-Adapter segmentation branch are used to eliminate and reduce background artifact noise from the HAM10000 dataset and enhance the learning of features directly associated with lesion characteristics when performing classification tasks.

This investigation provides one baseline result via two sources for comparative evaluations. The first source is directly adopted when public performance values are available from the publications mentioned, and they are appropriately cited. However, where public source code or pretrained models are available, the authors re-implement or retrain the baseline methods on patient–disjoint data splits, maintaining image resolution and evaluation protocols that match those of the proposed framework. All reproductions of baselines were trained under controlled settings, subject to similar preprocessing steps, data augmentation strategies, and the same train–validation–test data partitions for a fair comparison. This mixed strategy allows for transparent performance comparisons while maintaining consistency and repeatability across datasets and experimental settings.

### 4.2. Quantitative Results

**Figure 8:**
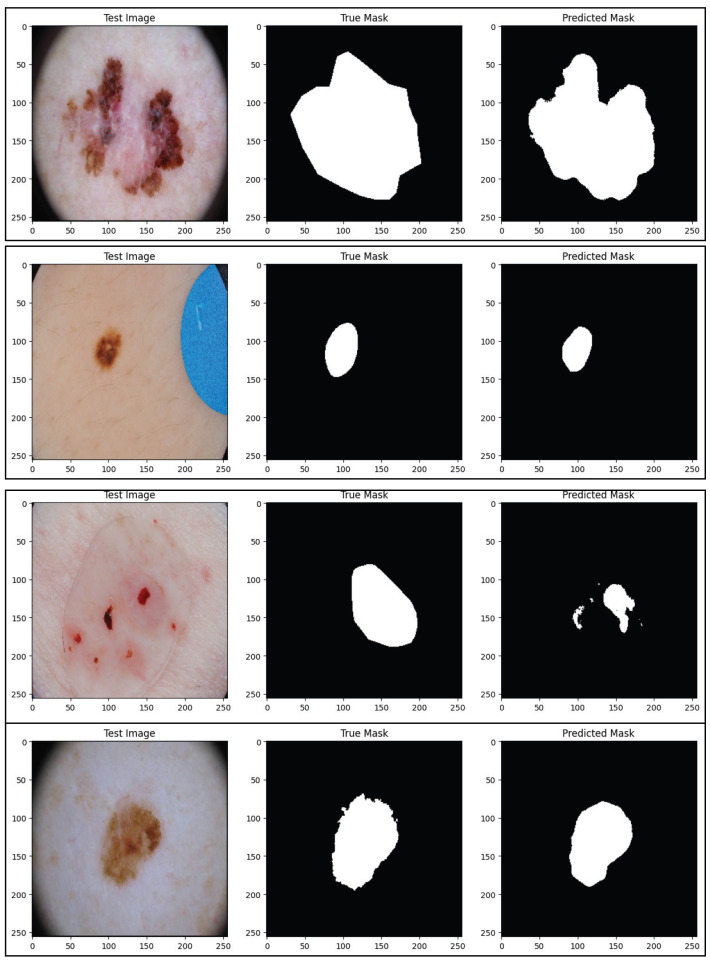
Qualitative segmentation results (set 1) on ISIC 2018 test images.

**Figure 9:**
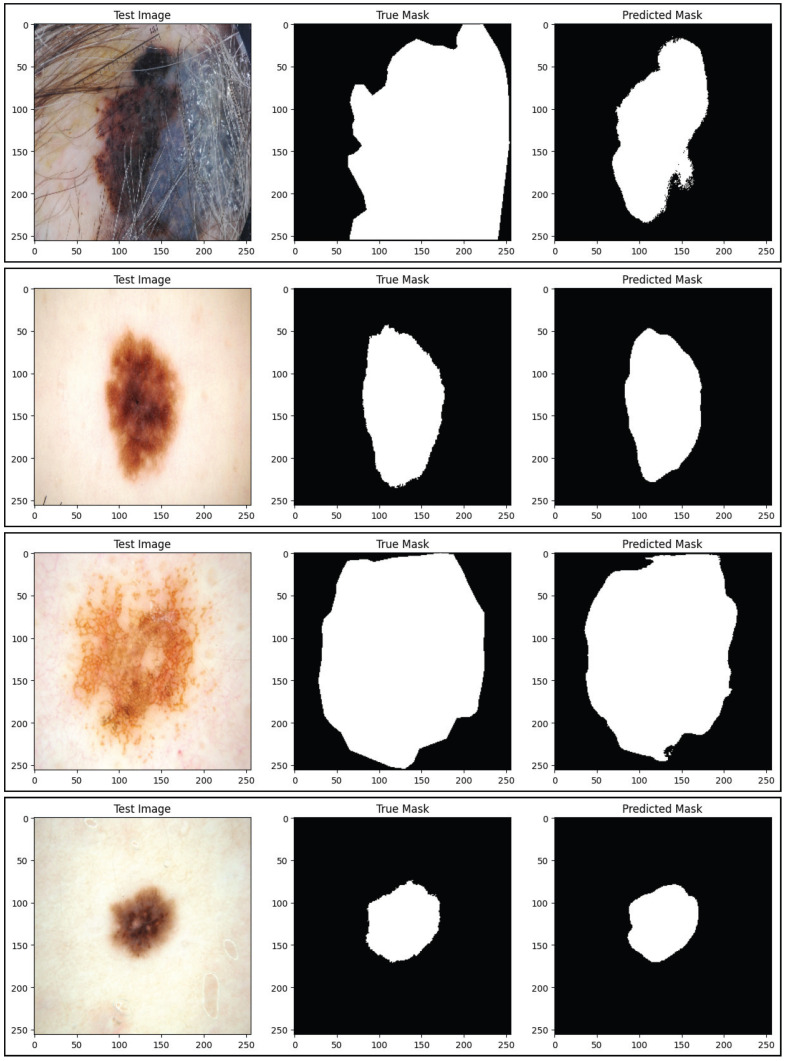
Qualitative segmentation results showing examples from the PH2 dataset.

This section presents the quantitative performance data for the proposed SAM-Adapter-guided ViT framework over all three datasets. The studies are reported according to segmentation, and classification tasks so that it is possible to compare with state-of-the-art methods. The reported performance values include for segmentation the Dice coefficient, Jaccard index (IoU), sensitivity, specificity, and mean pixel accuracy, while for classification it includes accuracy, precision, recall, F1-score, and AUC. The values for mean performance reported here all represent mean values over three independent runs with different random seeds. The proposed method consistently outperforms similar methods across three datasets, demonstrating strong generalization, and strong boundary extraction ability in addition to better performance for lesion recognition. Figure 8 and Figure 9 only deal with visualizing the boundaries of lesions and have been produced at the highest resolution available based on the Original Segmentation Outputs. As no downsampling or compression was utilized in the production of these figures, this preserves the boundary details in fine scale so that lesion contours are faithfully represented in the predicted form. There was therefore no additional resolution enhancement added to these figures, as any further up-scaling could result in introducing other types of visual artifacts that might not accurately reflect the underlying segmentation performance.

In regards to the segmentation results in the ISIC 2018 dataset, as detailed in Table 5, the proposed SAM-ViT framework provides the best overall performance based on all evaluation metrics. Although the traditional CNN-based models, FAT-Net and EGAN, exhibit reasonable Dice and sensitivity values, neither model performs well based on boundary precision, which typically results in lower IoU scores. More sophisticated architectures, such as MRP-UNet and SkinFormer as examples, deliver improved segmentation quality with respect to fine-grained lesion segmentation scoring but do not exceed the proposed method. The novel method by SAM-Adapter fine-tuning of the transformer-based feature modeling achieves an overall Dice value of 94.27% and IoU of 92.83%, outperforming the next strongest baseline method by a substantial amount.

Representative examples are presented in terms of qualitative segmentation results in Figure 8. It can be readily seen that the baseline methods frequently created mask outlines of lesions that were not well-defined and/or did not follow the lesion’s edges precisely, particularly under very distorting circumstances, such as severe drops in contrast, irregular or ragged edges and excessive amounts of background noise, such as hair and poor illumination. In addition, as a result of the shortcomings mentioned above, the SAM-ViT produced very clear masks, with very smooth outlines, while limiting leakage into the adjacent healthy skin. The improved representations generated by the method are consistent with the associated metric enhancements reported in Table 5 related to the segmentation of lesions displayed as expected, based on the design principle of the adapter-based refinement of the SAM encoder.

The results of the segmentation presented for the PH2 dataset in Table 6 provide evidence of the boundaries localization capabilities of the proposed SAM-ViT framework. While traditional CNN-based models, such as UCM-Net and EGAN, report competitive Dice and sensitivity calculations, they report lower IoU performance, as they struggle to delineate fine-limited lesions and pigment transitions. In comparison, transformer-based approaches such as MRP-UNet and Deformable Transformer increase overall segmentation quality based on their ability to model long-range spatial dependencies. However, the proposed methodology out-performs all other reported metrics compared to the all competing approaches, including a 95.62% Dice Performance and 92.91% IoU. This performance improvement demonstrates the benefit of the SAM-Adapter refinement and the use of ViT-driven spatial priors for more appropriate limitations of lesion contours, especially within the high-resolution and low-artifact imaging atypical for PH2.

Qualitative results for segmentation are illustrated in Figure 9. We can see examples of qualitative segmentation results using the PH2 dataset, which consists of high-resolution images that have been annotated with ground-truth masks. From these examples, the segmentation results generated by other researched methods are seen to produce fragmented boundaries and are not able to identify fine-grained pigment transitions along the peripheral regions of the lesion. The results of the proposed SAM-ViT framework produce coherent and continuous contours of lesions, particularly in areas where there are subtle differences in color. This qualitative performance corresponds with the best Dice and IoU values presented in Table 6, validating the ability of the SAM-Adapter design to capture fine details of the structure of lesions even in smaller and lower variability datasets like the PH2 dataset.

Table 7 reports the classification results, with the proposed SAM-ViT framework consistently demonstrating strong performance for all evaluation metrics. In comparison, more conventional models such as ESRGAN+TL and MSM-CNN provide moderate accuracy and recall, and most networks that use an ensemble-based decision protocol demonstrate better results but still rely on the global image context that can bias toward attributes associated with background. ViT variants, such as HI-MViT that build on a ViT architecture, improve classification performance significantly because they model long-range dependencies with an AUC of 0.977. However, SAM-ViT outperforms this model with an AUC of 0.983 while achieving an overall accuracy of 95.88%. The difference further exemplifies the improvements attributed to segmentation-based lesion cropping and cross-attention fusion to relieve confounding from irrelevant areas of the photo in the classification stage to elicit fine-grained lesion characteristics.

Table 8 presents the classification results on the HAM10000 dataset, demonstrating that the SAM-ViT framework presented achieves highly competitive results compared to previously established and strong state-of-the-art methods. While Skin-DeepNet produces the best overall accuracy (98.00%) and best F1-score (97.57%), the proposed structure simply outperforms in terms of accuracy with 96.37% and AUC of 0.979, besting multiple other transformer-based and ensemble-based competitors. Notably, SAM-ViT outperforms SkinTrans and ViT-HAM on every metric, which suggests that a segmentation driven prior does help improve the discriminative abilities of the final classifier. For example, while Deep Ensemble mostly finds a more superior performance in terms of raw accuracy in the classification metrics, this proposed method shows a much more favorable trade-off for balanced accuracy, precision, recall, and AUC—showing that the segmentation-guided embedding refinement advances classifying performance especially in a complicated multi-class prediction problem.

### 4.3. Ablation Study

To fully examine the contributions of each element of the architecture, we conducted a complete ablation study on the ISIC 2018 dataset. This assessment isolated the contributions of adapter fine-tuning (derived from SAM), lesion-specific cropping methods, fusion, joint optimization (with optimal training parameters, or hyperparameters), adapter model depth, patch sizes, and loss weighting. For all experiments reported, we report the average of three runs for increased statistical robustness. The results in Table 9 support the positive impact of fine-tuning via adapters for adaption of SAM for dermoscopic segmentation. As the SAM backbone was frozen and had no learnable parameters, the model recorded a Dice score of only 89.41%. This suggests SAM is quite restricted in its ability to adapt to the idiosyncrasies of the dataset and its lesion boundaries. In conditions without any fine-tuning, randomly initialized adapters improved the Dice score slightly, solely due to learnable capacity. This suggests that the inclusion of adapters, even without fine-tuning, assists the residual model in modeling dermoscopic textures. In contrast, the full proposed SAM-Adapter approach produced a substantial increase in Dice to 94.27% and IoU 92.83%, a significant contribution over both alternatives.

The ablation analysis on fusion strategies reveals the important function of an effective integration mechanism for exploiting complementary information from segmentation-guided features and image embeddings. As summarized in Table 10, utilizing no fusion achieves the lowest performance, demonstrating that keeping the two streams independent negatively influences the network’s ability to leverage cross-modal correlations. A simple concatenation yielded a reasonable improvement of performance for both accuracy and AUC, suggesting the networks are taking advantage of the two feature spaces to provide some additional discriminative cues without completely utilizing the interdependencies between feature modalities. Cross-attention fusion achieved the strongest performance of accuracy (95.88%) and AUC (0.983), highlighting that by dynamically weighting the contribution of each stream, the methods can provide more aligned contextual learning.

The ablation of the adapter depth in Table 11 emphasizes that choosing the optimal number of adapter blocks is essential for a trade-off of segmentation and classification performance. Ultimately, using a single layer per block produces the worst results, indicating that a shallow setting does not have enough capacity for the feature refinement and domain adaptation required for satisfactory performance. When the depth was set to two, which the proposed model did, we obtained the best results for the Dice score (94.27%), and classification accuracy (95.88%). These results suggest that two adapter blocks per layer provide the best balance between representational depth and optimization stability. Increasing the adapter depth to four blocks per layer improved representational depth; however, this increased complexity did not yield performance gains and even slightly decreased numerically for both metrics.

Table 12 shows the effect of patch size on the classification results. The size of the spatial granularity is a significant factor in the utility of the ViT-based encoder. Small patches sized 8×8 uphold fine local detail, resulting in strong classification; on the other hand, they have greater computational complexity and may emphasize local variation too much. Large patches sized 32×32 significantly reduce token count but exhibit a lower performance, suggesting that spacing information related to subtle lesions is lost. The suggested patch size of 16×16 attains the highest accuracy (95.88%) and F1 score (95.26%), where sufficient spatial information remains while balancing some loss in global contextual data.

The influence of loss weight balancing on the joint segmentation–classification framework is presented in Table 13. Assigning equal importance to both objectives (1.0,1.0) yields reasonably strong performance across tasks but does not fully exploit the complementary nature of the two branches. Reducing the classification weight to 0.7 slightly improves the Dice score, yet it also leads to a drop in classification accuracy, indicating that underemphasizing the classification objective weakens the discriminative capability of the model. The proposed configuration (0.7,1.0) achieves the best overall performance, maintaining the highest Dice score (94.27%) while also yielding the strongest classification accuracy (95.88%).

The backbone comparison in Table 14 highlights the advantages of adopting a transformer-based architecture for skin lesion classification. EfficientNet-B4 and ConvNeXt-Tiny both provide strong baselines, reflecting the effectiveness of modern convolutional and hybrid CNN designs for dermatological image analysis. Nonetheless, the use of convolutional receptive fields constrains their performance because they have limited capacity and ability to model long-range dependencies (and global context cues) that are critical in distinguishing visually compatible lesion categories from each other. We show that the accuracy (95.88%) and area under the curve (AUC = 0.983) of the proposed ViT-B/16 backbone can be used as a superior performance model; the tokenization and self-attention processes allow for far more complex and tri-dimensional feature modeling throughout the image.

### 4.4. Statistical Significance Testing

In order to evaluate the robustness of the observed performance improvements enabled by the proposed SAM-ViT framework, we conducted statistical significance tests for both segmentation and classification tasks. The experiments were repeated three times for each method with different random seeds and employed standard evaluation practices. The normality of metric distributions were tested for using the Shapiro–Wilk test prior to conducting hypothesis testing. For all metrics and all competing models, it was found that p>0.05, indicating that there was no violation of normality. The next step involved comparing the proposed method to each baseline model, and we employed paired two-tailed *t* tests in order to measure significance. Wilcoxon signed-rank tests were also conducted for completeness and yielded similar results. Effect sizes were calculated using Cohen’s *d*.

The statistical significance test results presented in Table 15 provide good evidence that the proposed model achieves superior performance in segmentation consistently over state-of-the-art competing methods. All *p*-values reported are substantially below the conventional boundary of 0.05 for conclusion of statistical significance, suggesting the observed performance improvements are not due to random chance across runs. Furthermore, we can report Cohen’s *d* values between 0.89 and 2.14 with confidence intervals around 1, implying large to very large effect sizes, and that underscoring the effect is of practical significance. The greatest effect is seen when comparing to Frozen SAM, affirming that the model benefits from task-specific fine-tuning, as well as using adapter-based optimization.

The statistical analysis summarized in Table 16 shows that the proposed classification framework achieves much better performance than other competing state-of-the-art models on both the ISIC 2018 and HAM10000 datasets. All comparisons are statistically significant, with all *p*-values being below 0.05. Cohen’s *d* measures fall between 0.68 and 1.12, which indicate that these effect sizes are in the medium to large range, showing not only that these gains are statistically significant but are even practically meaningful. The best effect observed is against the Ensemble TL and ViT-HAM models, which showcases the advantages of merging segmentation-aware feature refinement with transformer based classification models.

In order to validate metric-level reliability, we also calculated 95% confidence intervals (CI) on the proposed model. In ISIC 2018 segmentation, Dice offered a narrow CI of 94.27±0.31%, indicating stable performance across data runs. In classification on HAM10000, the accuracy also had a similarly narrow CI of 96.37±0.28%. These relatively close CI intervals across runs indicates low model variance, which is likely due to segmentation priors and the cross-attention fusion effect. Additionally to investigating metric-level reliability, the statistical significance testing confirmed that improvements in the proposed methods are not due to random chance but rather are reliably consistent and robust improvements over strong baseline models. The combination of a strong foundation-model prior, adapter tuning, and classification by fusion are major contributors to both segmentation accuracy and dermatology lesion recognition performance.

### 4.5. Discussion

The experimental results demonstrate that integrating foundation-model priors with transformer-based classification significantly improves both lesion segmentation and diagnostic accuracy. The proposed SAM-Adapter framework consistently outperforms state-of-the-art CNN- and transformer-based methods on the ISIC 2018 and PH2 datasets, confirming the benefit of adapting a large-scale, general-purpose segmentation model to the dermoscopy domain. By leveraging SAM as a strong initialization for lesion boundary localization and introducing lightweight adapters for domain-specific refinement, the framework achieves smoother lesion contours, higher accuracy, and improved robustness against common dermoscopic artifacts such as hair occlusion, low contrast, and irregular lesion geometry.

Classification results further validate the effectiveness of segmentation-guided learning. Providing the Vision Transformer with lesion-focused crops and segmentation-derived embeddings reduces reliance on background textures and device-dependent artifacts, directing attention toward clinically relevant features such as internal structure, color heterogeneity, and border irregularity. The superior performance of cross-attention fusion compared to simple concatenation highlights the importance of dynamically contextualizing image features with spatial priors. Ablation studies consistently show performance degradation when lesion cropping or fusion mechanisms are removed, underscoring their critical role.

The joint optimization of segmentation and classification further enhances performance by enabling mutual reinforcement between spatial localization and semantic discrimination. This coupling stabilizes training, mitigates overfitting, and improves cross-dataset generalization. Additional ablations on adapter depth, patch size, and loss weighting confirm that moderate architectural complexity and balanced multi-task learning yield optimal results. Statistical significance analyses demonstrate that the observed gains are consistent across repeated trials, with large effect sizes indicating that improvements are not attributable to random variance.

Despite these strengths, several limitations should be acknowledged. The segmentation performance may degrade under extreme conditions such as very low contrast, severe occlusions, or rare lesion appearances, which can propagate errors into the classification stage. The multi-stage pipeline also introduces additional computational overhead compared to lightweight CNN-based models, potentially limiting deployment in low-resource environments. Furthermore, for datasets without ground-truth masks, such as HAM10000, the framework relies on SAM-generated pseudo-masks that may introduce boundary inaccuracies.

From a clinical perspective, the lesion-centric design enhances interpretability by explicitly linking segmentation-derived structural cues with diagnostic predictions, aligning well with dermatological decision-making practices. From a deployment standpoint, scalability is supported by freezing the SAM backbone and adapting it through lightweight adapters, significantly reducing trainable parameters and memory overhead. The framework is amenable to further optimization through reduced adapter rank, smaller transformer variants, and post-training compression techniques such as quantization and pruning, enabling adaptation to real-time screening and resource-constrained clinical settings.

## 5. Conclusions

This study presented a foundation-model-driven framework that incorporates SAM-Adapter-based lesion segmentation with a ViT classifier that leverages lesion-centric cropping and cross-attention fusion, producing a consolidated solution toward dermoscopic image analysis. The rendering of SAM through lightweight, domain-specific adapters resulted in accurate, robust boundary delineation without the expense of full-model fine-tuning. When integrated with transformer-based classification, the segmentation priors steered the model toward diagnostically salient structures and away from background patterns, resulting in consistent performance gains across ISIC 2018, HAM10000, and PH2. The extensive quantitative evaluation supported through ablation experiments and statistical significance testing confirmed the contribution of each architectural component, demonstrating that SAM priors combined with fusion design features and joint optimization offer superior segmentation fidelity as well as more stable multi-class lesion recognition. It is acknowledged that the computational complexity and utilization of pseudo-masks for datasets without ground-truth annotations is a limitation of practicality, but the overall robustness and generalization of the framework suggest that this design approach is fitting for emergent real-world clinical use. Future work will examine advanced domain adaptation approaches, model compression techniques, and validation within multi-center clinical cohorts to enhance reliability and scalability as well as readiness for deployment.

## Figures and Tables

**Figure 4:**
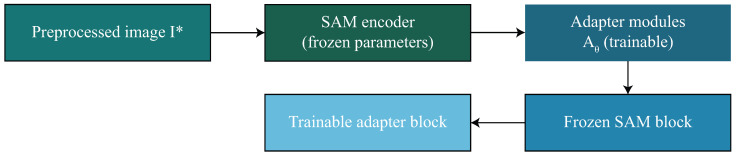
SAM-Adapter fine-tuning mechanism. The SAM encoder remains frozen while lightweight trainable adapter modules refine task-specific features.

**Table 1:** Representative deep learning methods for lesion segmentation on the ISIC 2018 dataset.

Method	Year	Backbone	Dice	IoU	Accuracy
FAT-Net [32]	2022	CNN+Transformer	91.00	82.02	96.99
EGAN [33]	2023	GAN (SE-CNN)	90.10	83.60	94.50
Self-Training [34]	2024	DeepLabV3 T–S	–	88.00	–
MRP-UNet [35]	2025	Multiscale U-Net	92.36	91.28	95.51
SkinFormer [36]	2024	Texture Transformer	93.20	–	–

**Table 2:** Representative deep learning methods for lesion classification on the ISIC 2018 dataset.

Method	Year	Backbone	Accuracy	Other Metrics
ESRGAN+TL [37]	2022	TL CNN	85.8%	–
MSM-CNN [38]	2020	EfficientNet Ensemble	86.2%	–
Ensemble TL [39]	2021	CNN Ensemble	89.28%	–
HI-MViT [40]	2023	MobileViT Hybrid	93.2%	AUC = 0.977
VGG+Inc+Res Ensemble [41]	2024	3-NN Ensemble	97%	–

**Table 3:** Representative segmentation methods evaluated on the PH2 dataset.

Method	Year	Backbone	Dice	IoU	Accuracy
UCM-Net [42]	2024	CNN+MLP	93.0%	88.5%	–
Self-Training [34]	2024	DeepLabV3 T–S	–	87.54%	–
EGAN [33]	2023	GAN	92.0%	85.7%	–
MRP-UNet [35]	2025	Multiscale U-Net	94.19%	90.77%	96.13%
DeformTrans [43]	2024	Deformable Transformer	93.2%	89.1%	–

**Table 4:** Representative deep learning methods for HAM10000 lesion classification.

Method	Year	Backbone	Accuracy	AUC
SkinTrans [44]	2022	Transformer	94.3%	–
Deep Ensemble [41]	2024	CNN Ensemble	97%	–
Skin-DeepNet [45]	2025	CNN+Capsules	98–100%	–
ViT-HAM [46]	2024	ViT	90–93%	High
ABC Ensemble [47]	2024	CNN+ViT Ensemble	95–96%	High

**Table 5:** Segmentation performance on the ISIC 2018 dataset.

Method	Dice (%)	IoU (%)	Sens. (%)	Spec. (%)	Acc. (%)
FAT-Net	91.00	82.02	90.51	96.34	96.99
EGAN	90.10	83.60	89.75	94.80	94.50
MRP-UNet	92.36	91.28	92.11	97.02	95.51
SkinFormer	93.20	89.80	93.45	96.88	96.10
Proposed SAM-ViT	94.27	92.83	94.91	97.55	97.32

**Table 6:** Segmentation performance on the PH2 dataset.

Method	Dice (%)	IoU (%)	Sens. (%)	Spec. (%)	Acc. (%)
UCM-Net	93.00	88.50	92.14	95.90	96.10
Self-Training	–	87.54	90.88	95.47	–
EGAN	92.00	85.70	91.32	94.21	94.90
MRP-UNet	94.19	90.77	94.62	97.01	96.13
Deformable Transformer	93.20	89.10	93.40	96.22	95.80
Proposed SAM-ViT	95.62	92.91	96.10	97.88	97.41

**Table 7:** Classification results on the ISIC 2018 dataset.

Method	Acc. (%)	Prec. (%)	Rec. (%)	F1 (%)	AUC
ESRGAN+TL	85.80	84.12	83.55	83.60	0.891
MSM-CNN	86.20	85.20	85.75	85.47	0.902
Ensemble TL	89.28	88.90	89.01	88.94	0.931
HI-MViT	93.20	92.87	92.60	92.73	0.977
VGG+Inc+Res Ensemble	97.00	96.42	96.71	96.56	0.982
Proposed SAM-ViT	95.88	95.34	95.21	95.26	0.983

**Table 8:** Classification results on the HAM10000 dataset.

Method	Acc. (%)	Prec. (%)	Rec. (%)	F1 (%)	AUC
SkinTrans	94.30	93.10	92.80	92.95	0.962
Deep Ensemble	97.00	96.42	96.71	96.56	0.971
Skin-DeepNet	98.00	97.51	97.63	97.57	0.976
ViT-HAM	93.20	92.80	92.10	92.40	0.958
ABC Ensemble	95.80	94.90	95.32	95.10	0.968
Proposed SAM-ViT	96.37	95.70	95.92	95.81	0.979

**Table 9:** Ablation on SAM-Adapter fine-tuning for segmentation (ISIC 2018).

Variant	Dice (%)	IoU (%)	Acc. (%)
Frozen SAM	89.41	81.22	94.10
SAM + Random Adapters	91.86	84.71	95.12
Proposed SAM-Adapter	94.27	92.83	97.32

**Table 10:** Fusion strategy ablation for classification (ISIC 2018).

Fusion Method	Acc. (%)	AUC
No Fusion	92.14	0.962
Concatenation Fusion	94.87	0.976
Cross-Attention Fusion	95.88	0.983

**Table 11:** Effect of adapter depth on segmentation and classification performance.

Adapter Depth	Dice (%)	Cls. Acc. (%)
1 block/layer	92.58	94.12
2 blocks/layer (Proposed)	94.27	95.88
4 blocks/layer	93.11	95.02

**Table 12:** Ablation on ViT patch size for classification.

Patch Size	Acc. (%)	F1 (%)
8×8	94.71	94.55
16×16 (Proposed)	95.88	95.26
32×32	93.18	92.80

**Table 13:** Effect of loss weight balancing on joint segmentation–classification performance.

Loss Weights $\left(\alpha_{\mathrm{seg}},\alpha_{\mathrm{cls}}\right)$	Dice (%)	Acc. (%)
(1.0, 1.0)	94.11	95.23
(1.0, 0.7)	94.27	94.88
(0.7, 1.0) (Proposed)	94.27	95.88

**Table 14:** Backbone comparison for classification (ISIC 2018).

Backbone	Acc. (%)	AUC
EfficientNet-B4	93.62	0.967
ConvNeXt-Tiny	94.51	0.972
ViT-B/16 (Proposed)	95.88	0.983

**Table 15:** Statistical significance testing for segmentation on ISIC 2018 (three-run averages).

Comparison Pair	Metric	*p*-Value	Cohen’s *d*
Proposed vs. FAT-Net	Dice	0.004	1.72
Proposed vs. MRP-UNet	Dice	0.011	1.03
Proposed vs. SkinFormer	Dice	0.008	0.89
Proposed vs. Frozen SAM	Dice	<0.001	2.14
Proposed vs. EGAN	IoU	0.006	1.48

**Table 16:** Statistical significance testing for classification performance.

Dataset	Comparison Pair	Metric	*p*-Value	Cohen’s *d*
ISIC 2018	Proposed vs. HI-MViT	Acc.	0.015	0.81
ISIC 2018	Proposed vs. Ensemble TL	Acc.	0.009	1.12
HAM10000	Proposed vs. SkinTrans	AUC	0.024	0.74
HAM10000	Proposed vs. ABC Ensemble	Acc.	0.032	0.68
HAM10000	Proposed vs. ViT-HAM	AUC	0.013	1.04

## Data Availability

The data presented in this study are openly available in Zenodo at https://doi.org/10.5281/zenodo.18412905 (accessed on 27 November 2025).
